# Nanowires Reinforce Oriented Macroporous Aerogels for Rapid Uranium Removal from Acidic Radioactive Wastewater

**DOI:** 10.1002/advs.76507

**Published:** 2026-07-28

**Authors:** Ze Liu, Simin Guan, Se Shi, Hao Wang, Wenya Zhou, Hui Wang, Tao Liu, Yihui Yuan, Ning Wang

**Affiliations:** ^1^ State Key Laboratory of Marine Resource Utilization in South China Sea School of Marine Sciences Hainan University Haikou P. R. China

**Keywords:** directional microchannels, nanowires reinforced, rapid rate, “reinforced concrete” structures, uranium removal

## Abstract

Rapid uranium removal from acidic wastewater is significant for nuclear pollution treatment. Robust mechanical strength is necessary for practical applications. Herein, we design a “reinforced concrete” structural aerogel (NWs/SP‐CSSB gel) with directional channels via nanowire‐assisted directional freezing. Benefiting from interconnected microchannels and hydrophilic ligands, the gel has ultrafast water permeability. Owing to the padding of ultralong nanowires (HAP NWs), the mechanical strength of the gel increased 70.43 times compared with that without NWs. Most uranium is removed within 2 min, and the removal ratio reaches equilibrium within 30 min in the highly acidic environment of pH 3. Meanwhile, it can reduce U to 9.75 ppb from the contaminated groundwater, achieving the drinking water discharge standard. Furthermore, the gel possesses good anti‐interference performance, even in a high‐salt environment and the presence of interfering ions, including lanthanides, radioelements, and transition metal ions; the U removal ratios of NWs/SP‐CSSB gel still maintain 84–96%. The adsorption mechanisms are carefully analyzed by using contrast experiments, kinetic and isotherm models, as well as FTIR and XPS spectra. Considering the high‐performance properties and simple fabrication of NWs/SP‐CSSB gel, we believe it has great potential in the application of radioactive wastewater treatment.

## Graphical abstract

A “reinforced concrete” structural aerogel with directional microchannels is engineered for rapid uranium capture. The interconnected architecture enables ultrafast water permeation, and the nanowire reinforcement yields a 70‐fold increase in mechanical strength. Efficient uranium removal is achieved within 2 min in acidic wastewater, and it exhibits robust performance in the presence of multiple interferences.

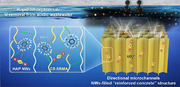

## 1 Introduction

As an important strategic resource, uranium plays a vital role in nuclear power generation and the nuclear fuel cycle. However, U is also a toxic radioactive element [1], and nuclear leakage is inevitable in uranium mining and nuclear power plant operation. Especially, acidic radioactive wastewater is usually generated because in situ leaching by using sulfuric acid is still the main method for uranium mining [2]. The half‐life of radionuclides is relatively long and poses a threat to the ecological and water environments [3, 4]. Therefore, the removal of U from acidic solutions is crucial for radioactive wastewater treatment. In addition, oriented microchannels and high mechanical strength are significant for the real application of adsorbents, because they can accelerate the solution transformation in the adsorbents and easily separate the used adsorbents from the purified wastewater [5, 6]. It is necessary to develop an adsorbent with a fast adsorption rate and high mechanical strength to treat acidic uranium‐containing wastewater.

UO_2_^2+^ is a Lewis acid, which is easy to form stable complexes with Lewis bases (such as carboxyl, phosphate, and amino) [7, 8]. Based on this theory, phosphate groups exhibit strong complexing ability with U(VI). In recent years, phosphate‐based chemicals have been effectively applied to the construction of fibers, polymers, and other adsorbents [9, 10, 11, 12]. However, these phosphate‐based adsorbents often lead to slow solution transport due to the disordered channels, which hinder the diffusion and mass transfer of UO_2_^2+^ inside the materials, and usually has a low adsorption rate of several hours and even 24 h [10, 13]. Therefore, constructing directional microchannels to accelerate the solution transportation in adsorbents is critical to improve the adsorption rate of uranium.

Using ice crystal template technology to control the nucleation and growth of ice crystals is an important strategy to construct directional porous structures [14]. In recent years, many materials with directional channels have been developed for uranium removal, such as PEI gels, PAO hydrogels, and PAO/GO hybrid sheet membranes [15, 16, 17]. However, due to the inherent drawbacks of directional freezing technologies, the mechanical strengths of the fabricated materials are very low, even as low as 2 KPa [18], and the adsorbents are fragile during the uranium removal and are difficult to separate from the purified wastewater.

Frame filling is an effective way to enhance the mechanical strength of gels [19]. The preparation of composite hydrogels by hybridizing organic/inorganic components has attracted more and more attention because it can endow the materials with better properties [20, 21]. As an emerging 1D inorganic material, ultralong hydroxyapatite nanowires (HAP NWs) have attracted more and more attention in the fabrications of flame‐retardant paper, biomaterials, energy and environmental materials due to their advantages in nanoscale diameter, high flexibility, abundant functional groups, and high biocompatibility [22, 23]. In addition, since the self‐assembly of HAP NWs can be adjusted in an orderly manner, the microstructure of fabricated gels by using HAP NWs can be conveniently controlled [24]. Therefore, HAP NWs provide a promising strategy for the construction of high‐strength gels.

**FIGURE 1:**
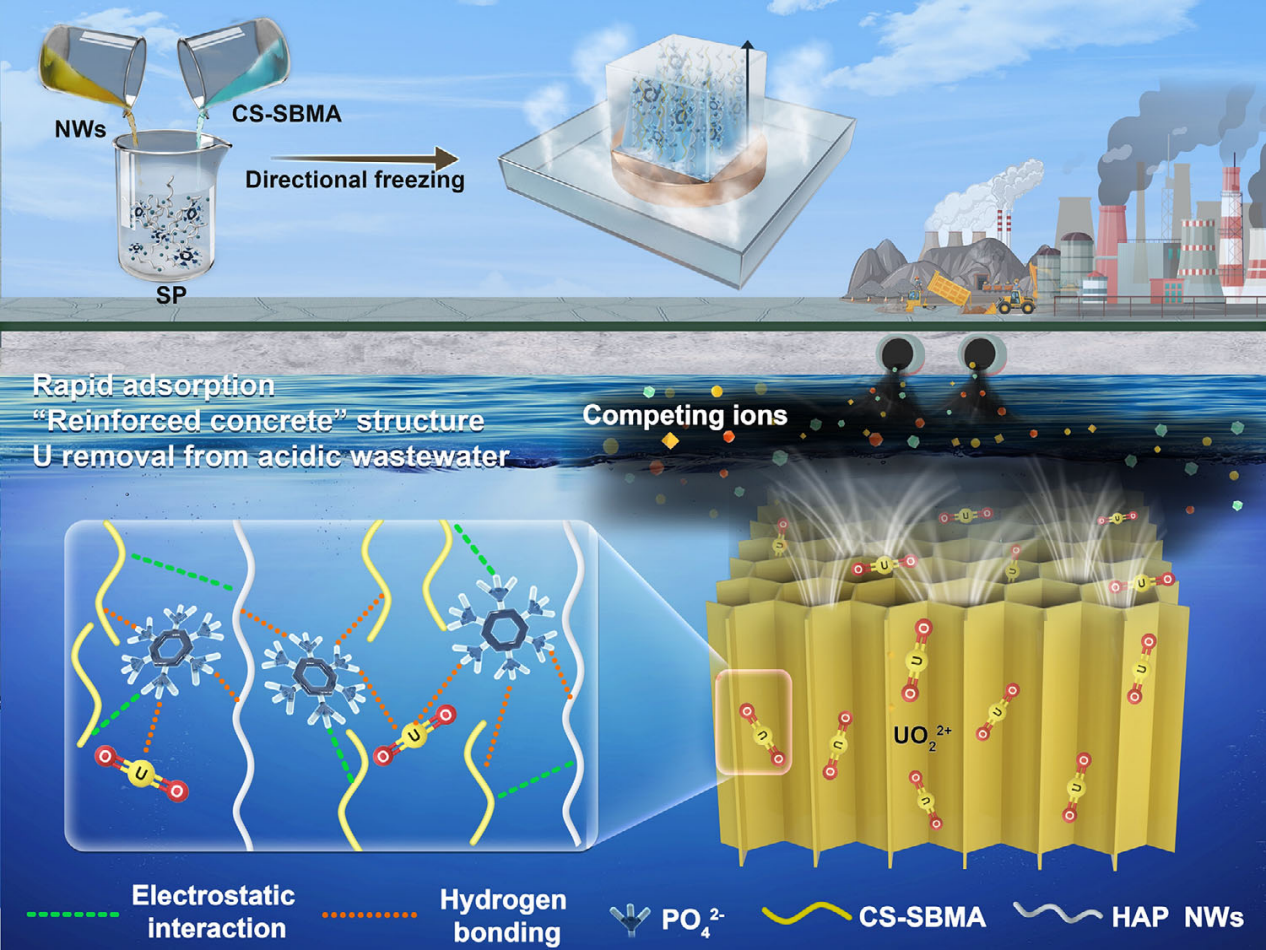
Schematic illustration for the fabrication and uranium removal of NWs/SP‐CSSB gels.

In this study, sodium phytate (SP) with six phosphate groups was introduced to enhance uranium chelation capacity and anti‐interference ability. The “reinforced concrete” structured NWs/SP‐CSSB gels with directional microchannels were prepared by using HAP NWs‐assisted directional freezing technology (Figure 1). The vertical‐microchannel structure was obtained, which was greatly beneficial to solution transport and uranyl ion diffusion, and resulting in an ultrafast uranium adsorption rate. The NWs/SP‐CSSB gels can remove most of the uranium in 2 min and reduce U to 9.75 ppb from the contaminated underground. The filling of HAP NWs improved the mechanical properties of gels, and the compression strength increased 70.43 times compared with that without NWs. In addition, in the interference of high salt, lanthanides, radioelements, transition metals, and common ions, the gels can still maintain high U adsorption efficiency of 84∼96%. We believe this gel has promising applications in radioactive wastewater treatment and uranium resource recovery.

## 2 Results and Discussion

### 2.1 Physicochemical Characterization of NWs/SP‐CSSB Gels

**FIGURE 2:**
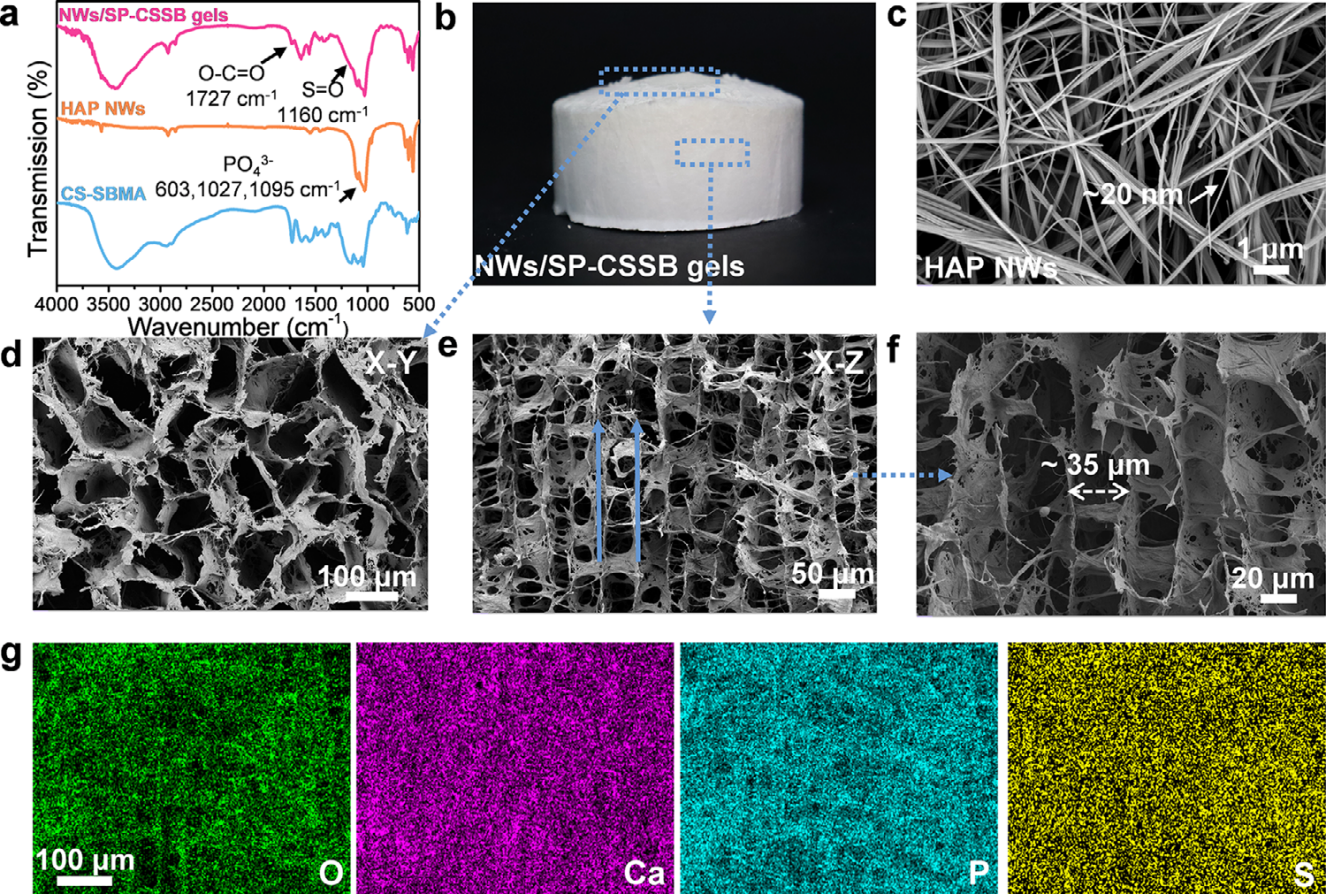
a) FTIR spectra of CS‐SBMA, HAP NWs, and NWs/SP‐CSSB gels, indicating the successful synthesis of these three materials. b) Photograph of the prepared NWs/SP‐CSSB gels. c) SEM image of HAP NWs showing their nanofiber microstructure. d‐f) SEM images of NWs/SP‐CSSB gels for the cross section (X‐Y plane) and vertical view (X‐Z plane). g) EDS mapping images of NWs/SP‐CSSB gels.

Besides the structural reinforcement effect of nanowires, the tight combination of the three components (NWs, SP, and CS‐SBMA) was attributed to their strong electrostatic interactions. In the fabrication of NWs/SP‐CSSB gels, NWs and SP were highly negatively charged due to the abundant phosphate groups, and the quaternary ammonium cations from CS‐SBMA can tightly combine NWs and SP via electrostatic attractions. Of course, hydrogen‐bond interactions also existed in the compounds, but the electrostatic attraction played the main role in the compounds. Herein, CS‐SBMA was synthesized via the ammonium persulfate‐initiated surface graft polymerization according to the references with some modifications [25]. The successful fabrication of NWs/SP‐CSSB gels (Figure 2b) was verified via FTIR and EDS spectra. As shown in Figure 2a, the characterizations of 2‐(N‐3‐Sulfopropyl‐N, N‐dimethyl ammonium) ethyl methacrylate (SBMA, C═O and S═O signals at 1727 and1160 cm^−1^, respectively) [25, 26] and HAP NWs (PO_4_^3−^ signals at 603, 1027, and 1095 cm^−1^) were present in NWs/SP‐CSSB gels, indicating the presence of CS‐SBMA (CSSB, copolymers of chitosan and SBMA) and nanowires in the gels. Meanwhile, the appearance of Ca element (characterizations of HAP NWs) in EDS mapping of gels also indicated the presence of nanowires in the fabricated gels (Figure 2g). In addition, in order to avoid signal interference from HAP NWs (also containing phosphate groups), the presence of SP in the gels was verified via the determination of the combination of SP and CSSB. Elemental mapping image and EDX spectra (Figure S1 and S2) of SP‐CSSB showed the appearance of P element (characterization of SP), verifying the presence of SP in the samples, which indicated the highly efficient electrostatic bonding of sodium phytate with CSSB, and the SP was bonded in the NWs/SP‐CSSB gels via the strong electrostatic interactions.

In addition, the grafting of SBMA on CS was necessary for the fabrication of gels because SBMA can endow CS with good water solubility and more positive charges, and the generated copolymer of CS‐SBMA was an important crosslinker in the construction of NWs/SP‐CSSB gels and can closely combine with SP and HAP NWs via electrostatic interactions. The successful polymerization of CS and SBMA was verified via the following phenomena: the characterization of SBMA appeared in the FTIR spectra of CS‐SBMA (Figure S3); the S element was present in the EDS mapping of CS‐SBMA (Figure S4); compared with CS, CS‐SBMA can be easily dissolved in deionized water (Figure S5a) and possessed a significantly improved hydrophilicity (Figure S5b).

The microstructures of NWs/SP‐CSSB gels were investigated by using SEM. As shown in Figure 2d, an ordered honeycomb structure was formed in the cross section (X‐Y plane). Specifically, in the vertical section (X‐Z plane, Figure 2e), benefiting from the filling of nanowires (∼20 nm in diameter and hundreds of micrometers in length, Figure 2c), NWs/SP‐CSSB gels showed directional channels by using HAP NWs as a support skeleton, which was similar to “reinforced concrete” structures. HAP NWs played the role of “steel” and CS‐SBMA played the role of “concrete”, enhancing the mechanical strength of NWs/SP‐CSSB gels. Furthermore, the diameter of the channels was ∼35 µm (Figure 2f); the directional microchannels accelerated the solution transport during the uranium removal.

The pore size was further determined via the N_2_ adsorption‐desorption experiments. Besides the micro‐sized macropores observed via SEM, the NWs/SP‐CSSB gels also possessed abundant micropores and mesopores (with a diameter of ∼10.33 nm, Figure S7). As a control, the SP‐CSSB gels (without nanowires) did not contain mesopores. These results indicated that the addition of nanowires improved the microstructures of NWs/SP‐CSSB gels, and the abundant mesopores can further accelerate the solution transport in the aerogels. In addition, the NWs/SP‐CSSB gels possessed an ultralight characteristic (Figure S8), and the high porosity of 96.3%, and a specific surface area increased by 66.34% owing to the introduction of NWs (Figure S9), indicating that NWs improved the microstructures of gels and enhanced the contact area with uranium solutions.

**FIGURE 3:**
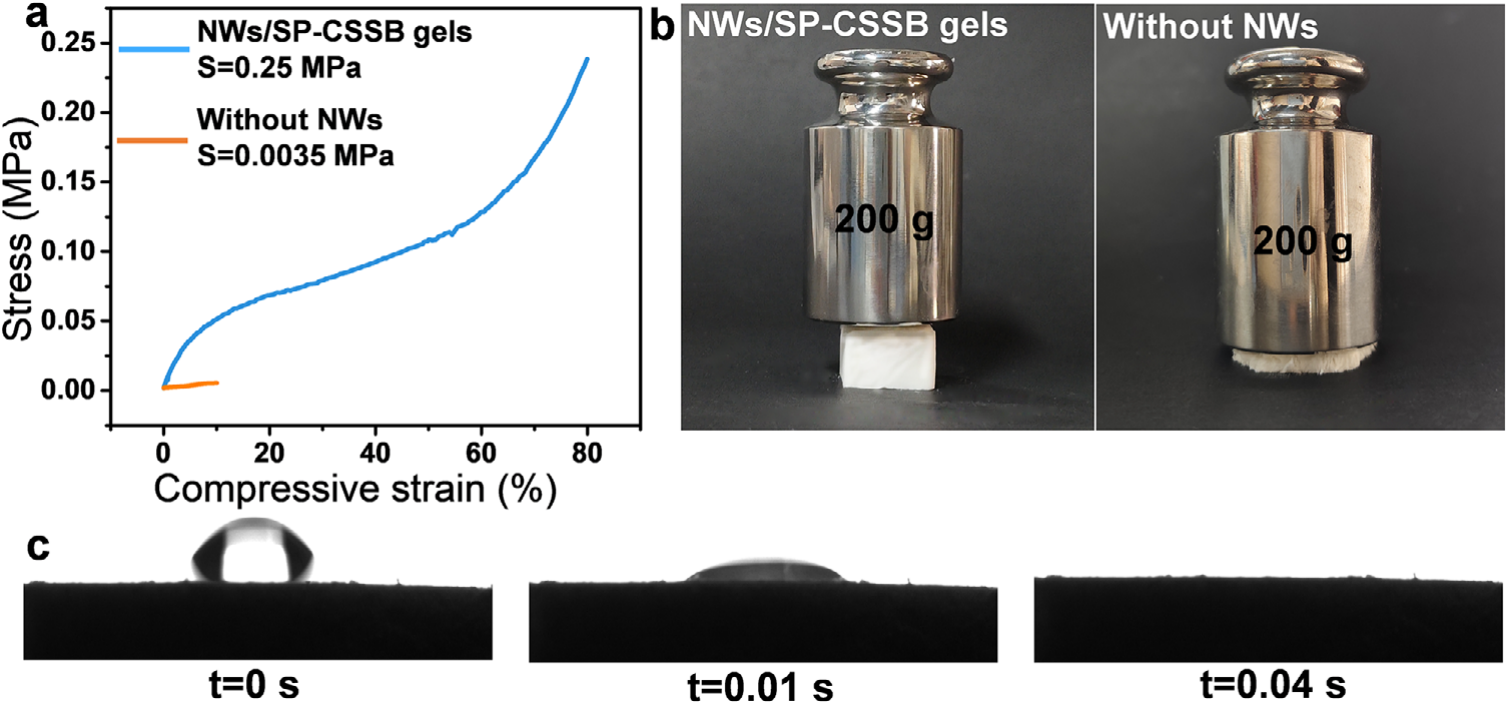
a, b) Stress‐strain curves and vertical compression tests of NWs/SP‐CSSB gels (with NWs) and SP‐CSSB gels (without NWs) to compare their mechanical properties. c) The sink rate of water droplets (deionized water) in NWs/SP‐CSSB gels to evaluate their hydrophilicity.

NWs largely enhanced the mechanical strength of NWs/SP‐CSSB gels due to their “steel” roles. This result was illustrated via the compression test. The NWs/SP‐CSSB gels possessed a much higher compression strength (0.25 MPa) compared with the gels without NWs (0.0035 MPa) (Figure 3a). The NWs/SP‐CSSB gels can withstand a 200 g weight without any damage and were intact after being immersed in solutions for a week (Figure 3b and Figure S10). However, the SP‐CSSB gels (without NWs) were broken after the compression and immersion test. The good mechanical strength of NWs/SP‐CSSB gels can be attributed to their “reinforced concrete” microstructures. As observed in the SEM image of NWs/SP‐CSSB gels, vertical nanowires were filled in the gels and played a powerful supporting role, just like “steel”. However, no “vertical steels” were observed in the SP‐CSSB gels (without NWs) (Figure S11). The “reinforced concrete” microstructures improve the mechanical properties of the NWs/SP‐CSSB gels.

The hydrophilicity of the adsorbent is a crucial factor influencing its uranium adsorption behavior [27]. To evaluate this, the water droplet penetration rates of three materials (NWs/SP‐CSSB gels, CS‐SBMA, and CS) were examined through dynamic contact angle (WCA) measurements. As shown in Figure 3c and Figure S5b, the water droplet completely penetrated the NWs/SP‐CSSB gel in just 0.04 s (WCA reached zero), while the penetration time for CS‐SBMA was around 0.08 s. In contrast, the water droplet penetration time for CS was significantly longer, approximately 1.96 s. Comparing the three materials, the order of hydrophilicity is NWs/SP‐CSSB gels>CS‐SBMA>CS. In addition, uranium solutions (pH 3, 12 ppm U‐spiked) can also quickly penetrate the gels (0.033 s, Figure S6), confirming that the NWs/SP‐CSSB gel exhibits superior hydrophilicity. In addition to the SBMA ligand, the excellent hydrophilicity of NWs/SP‐CSSB gels is further attributed to the 3D channel structure supported by HAP NWs.

### 2.2 Uranium Removal Performances

#### 2.2.1 Effects of pH and Adsorbent Dosage on Uranium Adsorption

The dosage of adsorbent is a key factor for evaluating its application performance. The optimal adsorbent dosage was studied via a series of adsorption experiments. As shown in Figure S13, the uranium adsorption efficiency of NWs/SP‐CSSB gels increased with the adsorbent dosage. When the dosage reached 0.06 g L^−1^, the further increase of dosage only resulted in minimal change in sorption efficiency. Considering the economic cost, the dosage of 0.06 g L^−1^ is suitable for the adsorption experiments.

**FIGURE 4:**
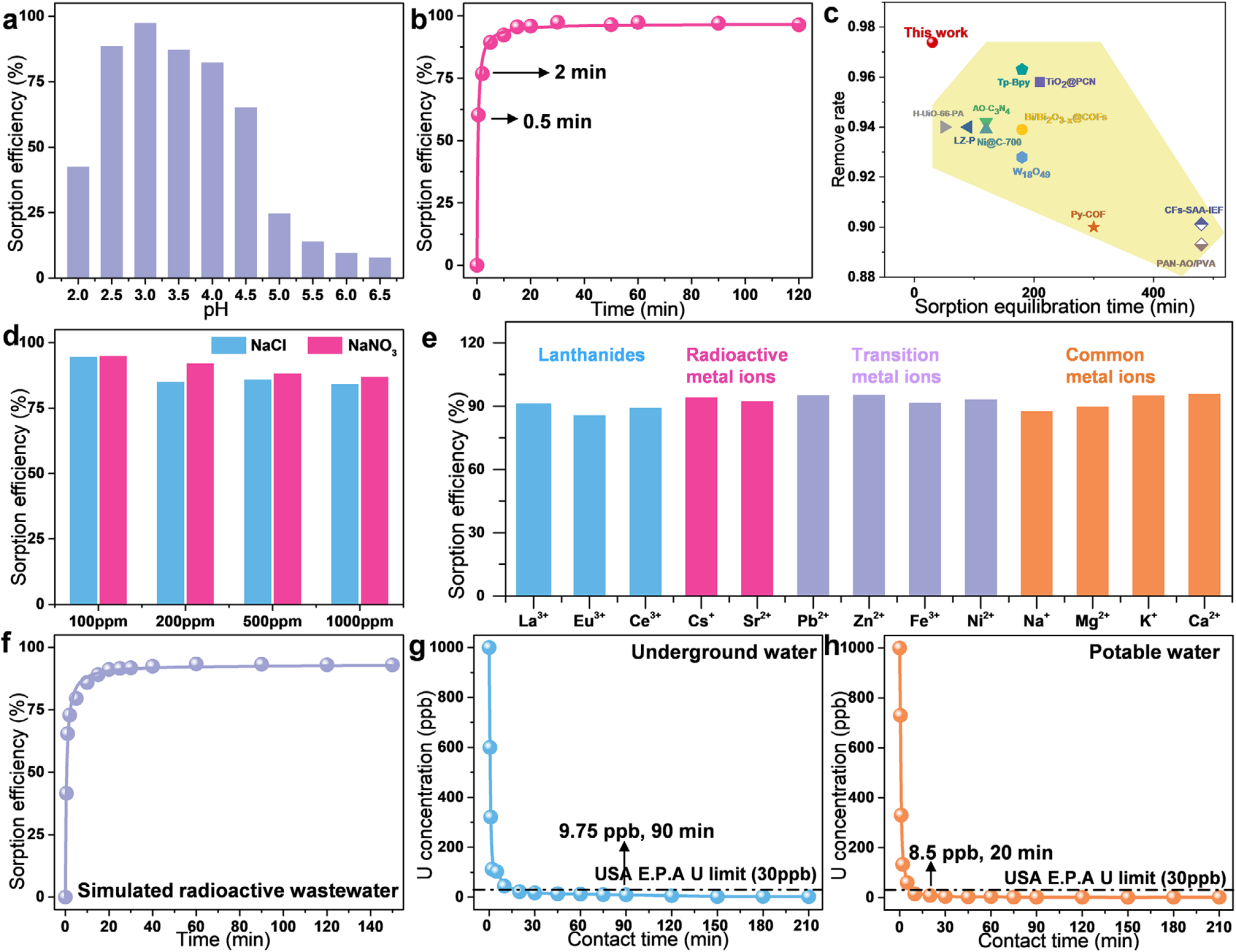
(a) Effect of pH on uranium adsorption (experimental conditions: C_0_ = 12 ppm, *m*/*V* = 0.06 g L^−1^, 306.15 K). b) Adsorption kinetics in uranium solutions (experimental conditions: C_0_ = 12 ppm, *m*/*V* = 0.06 g L^−1^, pH = 3, 306.15 K). c) The comparison of U uptake rate and capture capacity with other adsorbents. d) Effect of salt concentration on uranium adsorption (experimental conditions: C_0_ = 12 ppm, *m*/*V* = 0.06 g L^−1^, pH = 3, 306.15 K). e) Effect of coexisting ions (including in lanthanides, radioactive ions, transition metal ions, and common ions) on uranium adsorption of NWs/SP‐CSSB gels (experimental conditions: C_0_ = 12 ppm, *m*/*V* = 0.06 g L^−1^, pH = 3, 306.15 K). f) Uranium adsorption kinetics in simulated radioactive wastewater (experimental conditions: C_0_ = 18.9 ppm, *m*/*V* = 0.15 g L^−1^, pH = 3, 306.15 K). g‐h) Removal kinetics of uranium from different water samples by NWs/SP‐CSSB gels (*m*/*V* = 0.13 g L^−1^, pH = 3, 306.15 K).

pH affects the surface charge of NWs/SP‐CSSB gels and the form of uranium species, largely influencing the adsorption efficiency. The optimal pH of NWs/SP‐CSSB gels for uranium removal was evaluated in 12 ppm U‐spiked solutions within the pH range of 2 to 6.5 at the *m/V* of 0.06 g L^−1^. As shown in Figure 4a, the adsorption efficiency of NWs/SP‐CSSB gels increased from 42.48% to 97.4% as the pH increased from 2 to 3. Subsequently, the removal efficiency dropped as the increase of pH values. These results can be explained by the following reasons: different from the traditional PA (phytic acid)‐based adsorbents, NWs/SP‐CSSB gels were highly negatively charged owing to the introduction of sodium phytate. When pH≤3, U(VI) mainly existed in the form of UO_2_^2+^ [28]. However, when pH>3, UO_2_^2+^ usually hydrolyzed and formed uranium hydroxide complexes, resulting in a large increase in the effective ionic size of U species [29]. Furthermore, negatively charged U species will form when pH>5, largely weakening the electrostatic interactions between sodium phytate and uranium species. The NWs/SP‐CSSB gel can efficiently remove uranium (97.4%) at pH 3, highlighting its potential application in the removal of uranium from acidic radioactive wastewater. Based on these findings, subsequent experiments were conducted at pH 3.0. Besides adsorbent dosage and pH, the effect of solution temperature on uranium adsorption was also determined; the results indicated that the optimal temperature for uranium adsorption of NWs/SP‐CSSB gels was 306.15 K (Figure S12).

#### 2.2.2 Adsorption Kinetics

NWs/SP‐CSSB gels exhibited rapid uranium sorption efficiency compared to most of the reported adsorbents (Table S2) [30, 31, 32, 33, 34, 35, 36, 37, 38, 39, 40, 41, 42, 43, 44, 45, 46, 47, 48, 49, 50]. As shown in Figure 4b, the U (VI) adsorption efficiency of NWs/SP‐CSSB gels increased rapidly within the first 5 min, reaching adsorption equilibrium at 30 min, with the sorption efficiency achieving 97.39%. Significantly, it can remove most of the uranium in the first 2 min and has an ultrafast adsorption rate in the first 30 s. This result is superior to the recently reported adsorbents (Figure 4c) [30, 31, 37, 38, 39, 41, 42, 44, 45, 46, 47]. In addition, the gels exhibited good structural stability after the adsorption process (Figure S21). To further describe the adsorption kinetics, the pseudo‐first‐order and pseudo‐second‐order models were applied (Figure S14 and Table S3). The results indicated that the pseudo‐second‐order kinetic model (R^2^ = 0.999) provided a better correlation than the pseudo‐first‐order model (R^2^ = 0.429). This suggests that the adsorption behavior of NWs/SP‐CSSB gels is primarily governed by chemisorption, which is the dominant rate‐controlling factor. Furthermore, as shown in Figure S15 and Table S4, the maximum adsorption capacity of NWs/SP‐CSSB gels was 466.3 mg g^−1^, as determined by adsorption isotherms. The Langmuir model was found to adequately describe the adsorption process (Figure S16), indicating that the adsorption mechanism was predominantly monolayer chemisorption.

#### 2.2.3 Effects of Competing Ions on Uranium Adsorption

In environmental wastewater, high salt concentrations and various competing ions are commonly present. Therefore, the adsorption performance of NWs/SP‐CSSB gels was further evaluated in 12 ppm U‐spiked solutions with different salt concentrations, using NaCl and NaNO_3_ (100, 200, 500, and 1000 ppm) as the pollution templates. As shown in Figure 4d, the NWs/SP‐CSSB gel maintained high U (VI) adsorption efficiencies (94.46% and 94.87%, respectively) in NaCl and NaNO_3_ solutions at a salt concentration of 100 ppm. Even when the salt concentration increased to 1000 ppm, the adsorption rates remained at 84.12% and 86.94%, respectively, indicating that the NWs/SP‐CSSB gel exhibited remarkable salt tolerance under high salinity conditions.

Furthermore, as illustrated in Figure 4e, the NWs/SP‐CSSB gel demonstrated good uranium adsorption efficiency (86.06%∼96.19%) in the interfering ions, including in lanthanide (La^3+^, Eu^3+^, Ce^3+^), radioactive ions (Sr^2+^, Cs^+^), transition metal ions (Pb^2+^, Zn^2+^, Fe^3+^, Ni^2+^), and common ions (Na^+^, Mg^2+^, K^+^, Ca^2+^). The concentrations of interfering ions were set to 5 times (60 ppm), 2 times (24 ppm), 2 times (24 ppm), and 10 times (120 ppm) the initial uranium concentration (12 ppm), respectively. Meanwhile, the NWs/SP‐CSSB gel exhibited efficient uranium adsorption ability in the interferences of competing anions (Cl^−^, NO_3_^−^, NO_2_^−^, SO_4_^2−^, and I^−^, Figure S17). These results suggest that the NWs/SP‐CSSB gel maintained significant uranium adsorption potential even in complex wastewater containing multiple interfering ions. It is reported that PO_4_^2−^ exhibits strong affinity with UO_2_^2+^ based on the Lewis acid‐base theory; UO_2_^2+^ is more likely to accept electrons compared with other ions due to its high charge/radius ratio, and the stable coordination bonds (between lone pair electrons of PO_4_^2−^ and empty orbital of UO_2_^2+^) are formed during the adsorption [34, 51, 52]. Therefore, the NWs/SP‐CSSB gels can still exhibit good U (VI) capture ability in the multiple interferences.

#### 2.2.4 Uranium Removal From Simulated Radioactive Wastewater and Real Water Samples

To assess the practical application potential of NWs/SP‐CSSB gels in treating uranium‐contaminated wastewater, experiments were conducted using simulated radioactive wastewater (Table S1). As shown in Figure 4f, in the simulated radioactive wastewater with a uranium concentration of 18.9 mg L^−1^, they achieved a high removal efficiency of 93.34% within 60 min, indicating the practical application potential of NWs/SP‐CSSB gels.

Furthermore, potable water and groundwater were collected and used in the adsorption experiments. Herein, 39 mg of adsorbents were added to 300 mL of 1000 ppb uranium‐contaminated samples. The residual uranium concentration in the contaminated water samples was analyzed by ICP‐MS. As shown in Figure 4g,h, NWs/SP‐CSSB gels can reduce U to 8.5 ppb and 9.75 ppb from the contaminated potable water and groundwater within 20 and 90 min, respectively. These removal levels were well below the drinking water discharge standard of 30 ppb set by the U.S. Environmental Protection Agency (EPA).

### 2.3 Adsorption Mechanism

**FIGURE 5:**
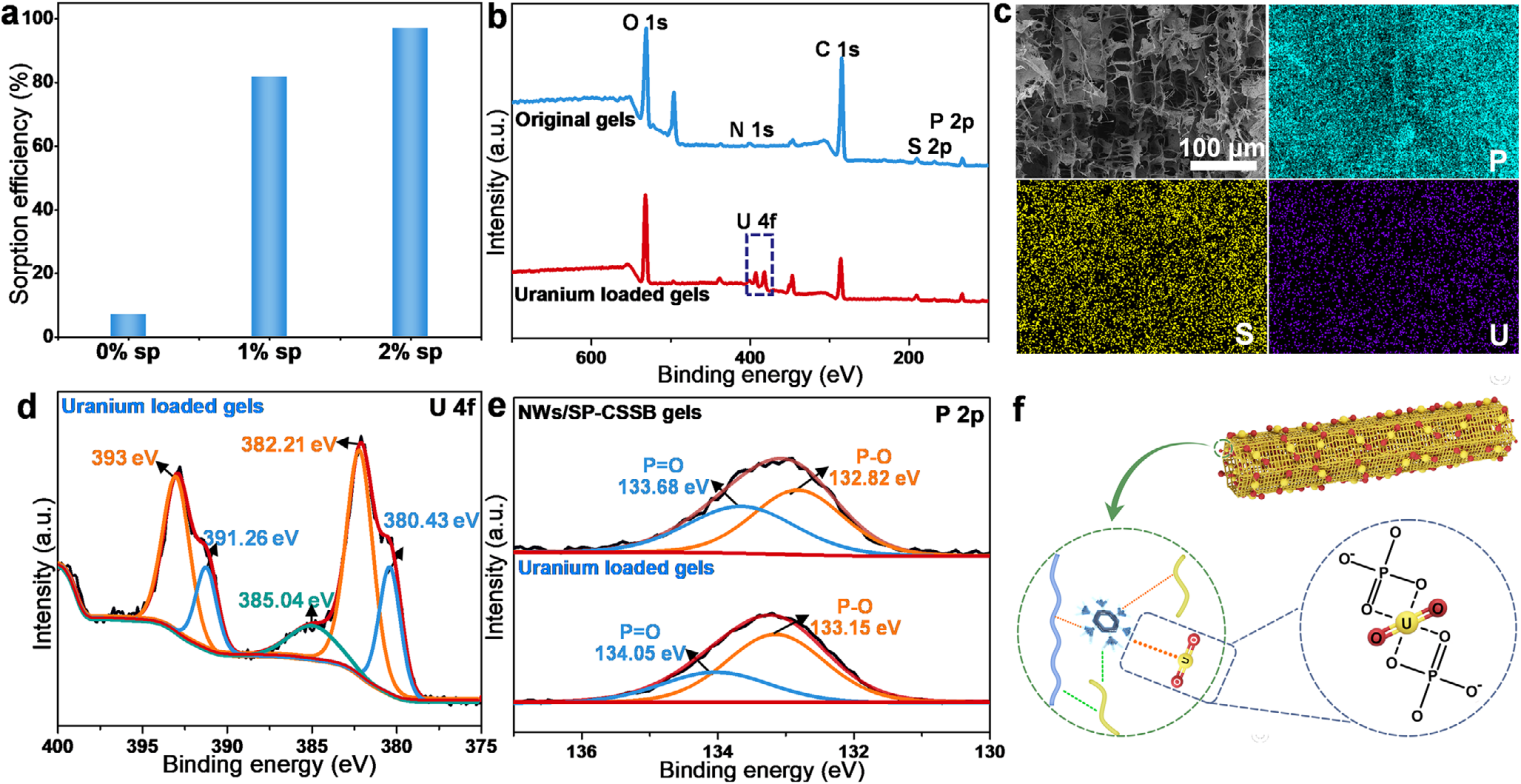
Mechanisms of uranium adsorption by NWs/SP‐CSSB gels. a) Effect of different SP contents on uranium adsorption capacities. b) XPS spectra of the NWs/SP‐CSSB gels and the U‐loaded gels. c) EDS elemental mapping of U‐loaded NWs/SP‐CSSB gels. d) High‐resolution XPS spectrum (in U 4f area) of U‐loaded NWs/SP‐CSSB gels. e) High‐resolution XPS spectra (in P 2p area) of NWs/SP‐CSSB gels before and after uranium adsorption. f) Bonding mechanism of uranyl ions on the prepared gels.

First, the main chemical component for U adsorption was confirmed to analyze the adsorption mechanisms. Herein, a series of adsorbents with varying SP contents (0%, 1%, and 2%) were prepared, and their uranium adsorption capacities were compared. As shown in Figure 5a, the NWs/CSSB gel (without SP) exhibited a uranium removal efficiency of only 6.88%. The uranium removal efficiency increased with SP content. When the SP content was increased to 2%, the adsorption efficiency of gels for uranium increased significantly to 97.39%. These results indicate that the PO_4_^2−^ group provided by SP played key roles in uranium adsorption for NWs/SP‐CSSB gels. It is reported that phosphate groups act as coordination sites and usually form stable chelates with UO_2_^2+^ through the P═O and P─O─H functional groups [53, 54].

Second, the chemical compositions of gels before and after uranium adsorption were compared by using FTIR and EDS to confirm the adsorption. As shown in the FTIR spectra, the characterization of UO_2_^2+^ (at 916 cm^−1^) appeared in the U‐loaded NWs/SP‐CSSB gels (Figure S18). In addition, after uranium adsorption, characteristic peaks of U 4f appeared in the XPS spectra, which could be deconvoluted into two distinct peaks corresponding to U 4f_5_/_2_ (393 eV) and U 4f_7_/_2_ (382.21 eV) (Figure 5b,d). Additionally, the color change (Figure S19) and the appearance of the U element in EDS mapping and EDX spectra (Figure 5c; Figure S20) for NWs/SP‐CSSB gels further supported the adsorption of U(VI).

Third, XPS spectra (in P 2p and S 2p areas) of NWs/SP‐CSSB gels before and after uranium adsorption were compared to confirm the coordination sites. Compared with the high‐resolution P 2p spectra before and after uranium adsorption (Figure 5e), the signal shifts for P═O bond (from 133.68 to 134.05 eV) and P─O (from 132.82 to 133.15 eV) were observed, indicating the involvement of PO_4_^2−^ groups in the coordination of UO_2_^2+^ (the detailed bonding mechanism was shown in Figure 5f). In the high‐resolution S 2p spectra (Figure S22), the peaks for S 2p_1_/_2_ and S 2p_3_/_2_ shifted positively by 0.26 and 0.16 eV, respectively, suggesting that the SO_3_^−^ groups also took part in the coordination of uranyl ions. These phenomena were consistent with the reports that PO_4_^2−^ groups can largely adsorb U(VI) and SO_3_^−^ groups can promote the adsorption process [52, 55].

## 3 Conclusions

In summary, a novel “reinforced concrete” structural gel (NWs/SP‐CSSB gel) with oriented microchannels was developed by using nanowires‐assisted directional freezing technology. The extremely fast water droplet penetration rate (0.04 s) of gels was attributed to the presence of directional microchannels and hydrophilic ligands. Moreover, the incorporation of HAP NWs significantly improved the mechanical strength of NWs/SP‐CSSB gels, and the strength values increased 70.43 times. In acidic solutions of pH 3, the uranium removal efficiency reached 97.39% within 30 min, and the gels were capable of reducing the uranium concentration to 9.75 ppb in the contaminated groundwater within 90 min. The maximum adsorption capacity was 466.3 mg‐U/g‐Ads. The adsorption process followed pseudo‐second‐order kinetics and Langmuir isotherm models. Even in the presence of high salt concentrations and a variety of competing ions, including in lanthanides, radioactive ions, transition metals, and common ions, the NWs/SP‐CSSB gel still maintained efficient uranium removal (84–96%). The adsorption mechanisms were further confirmed through FTIR and XPS analyses. Overall, the NWs/SP‐CSSB gel exhibited excellent mechanical stability, high adsorption performance, and a straightforward preparation process. Considering its outstanding performance and facile fabrication, this material offers a promising and cost‐effective solution for uranium removal from radioactive wastewater.

## 4 Experimental Section

### 4.1 Synthesis of NWs/SP‐CSSB Gels

First, the HAP NWs were synthesized by using a solvothermal method according to the previous report with slight modifications [56]. Details were shown in the Supporting Information.

Second, CS‐SBMA was synthesized via surface graft polymerization. Briefly, 0.5 g of CS was dissolved in 50 mL of acetic acid solution (1%) at 65°C. Then, 50 mg of ammonium persulfate was added with a nitrogen atmosphere. Finally, 1 g of SBMA was slowly added to the mixture, and the reaction continued for 8 h under an N_2_ atmosphere. The co‐polymers were subjected to dialysis and drying for further use.

Third, NWs/SP‐CSSB gels were obtained by using directional freezing technology. HAP NWs were dispersed in the SP aqueous solution to form a uniform suspension. Then, CS‐SBMA was dissolved in ultrapure water and was added to the mixture. Herein, the concentrations of the three components (NWs, SP, and CS‐SBMA) were all 20 mg mL^−1^. After stirring of 20 min, the mixture was poured into a PTFE circular mold, which was placed on a copper block, followed by a directional freezing process using liquid nitrogen. Finally, the samples were transferred to a freeze dryer and dried for 48 h to obtain NWs/SP‐CSSB gels. As a control, the NWs/CSSB gels (without adsorption ligand, SP) and SP‐CSSB gels (without reinforced materials, NWs) were also prepared by using the same method except that the SP and NWs were not added in the mixture, respectively, to investigate the roles of these three components.

### 4.2 Uranium Adsorption Ability Test

Effect of pH on uranium adsorption was evaluated in 12 ppm U‐spiked solutions. Herein, the pH values (from 2 to 6.5) of the solutions were adjusted by using 0.3 M HCl or NaOH, and 6 mg NWs/SP‐CSSB gels were immersed in 100 mL uranium solutions with different pH, respectively. After the adsorption equilibrium, the uranium concentration in the solutions was determined, and the uranium sorption efficiency (SE, %) was calculated by using the following formula.

$$SE\;\left(\%\right)=\frac{\left(C_{0}-C_{e}\right)}{C_{o}}\;\times 100\%$$

The optimal adsorbent dosage was investigated in 12 ppm U‐spiked solutions of pH 3 with *m/V* values ranging from 0.01 g L^−1^ to 0.09 g L^−1^. The adsorption kinetics were tested by dispersing 6 mg of adsorbents in a 100 mL uranium solution with a uranium concentration of 12 mg L^−1^. The kinetics parameters were calculated by using the following equations:

$$\ln\left(q_{e}-q_{t}\right)=\;\ln q_{e}-k_{1}t$$

$$\frac{t}{q_{t}}=\frac{1}{k_{2}{q_{e}}^{2}}+\frac{t}{q_{e}}$$

The maximum uranium capture capacity was tested by immersing 6 mg of adsorbents in 100 mL solutions (pH 3) with different uranium concentrations ranging from 2 to 50 ppm, and the adsorption isotherms were calculated by using the following models:

$$\frac{C_{e}}{q_{e}}=\frac{1}{q_{m}K_{L}}+\frac{1}{q_{m}}C_{e}$$

$$\ln\;q_{e}=\ln K_{F}+\frac{1}{n}\ln C_{e}$$

### 4.3 Effects of Competing Ions on Uranium Adsorption

Effect of salinity on uranium adsorption of NWs/SP‐CSSB gels was evaluated by measuring the U uptake capacity in high‐concentration NaCl and NaNO_3_ solutions (100, 200, 500, and 1000 ppm). In the test, 6 mg gels were immersed in 100 mL saline solutions, respectively, and 12 ppm uranium was added, after a stirring of 2 h, the uranium adsorption amounts of NWs/SP‐CSSB gels in these highly saline solutions were compared with that of solutions without interference.

In addition, effect of competitive interference ions on U uptake was carried out in U‐spiked solutions (pH 3) containing single interfering ions of 60 ppm lanthanides (La^3+^, Eu^3+^, Ce^3+^), 24 ppm radioactive metal ions (Cs^+^, Sr^2+^), 24 ppm transition metal ions (Pb^2+^, Zn^2+^, Fe^3+^, Ni^2+^), and 120 ppm common metal ions (Na^+^, Mg^2+^, K^+^, Ca^2+^), respectively. In the test, the *m/V* and the uranium concentration were 0.06 g L^−1^ and 12 ppm, respectively.

### 4.4 Uranium Removal From Simulated Radioactive Wastewater

Simulated radioactive wastewater was prepared according to the references [57], i.e., multiple interfering ions (Ba^2+^, Mg^2+^, Pb^2+^, Cr^3+^, Al^3+^, Mn^2+^, Co^2+^, Ni^2+^, Zn^2+^, Sr^2+^, Cu^2+^, Fe^3+^, Cd^2+^) (detailed information in Table S1) were added into an 18.9 ppm uranium‐contaminated solution. NWs/SP‐CSSB gels were immersed in the simulated radioactive wastewater at a *m/V* value of 0.15 g L^−1^, and the pH was adjusted to 3. After a certain contact time, some solutions were taken out to measure the residual uranium concentration, and the U sorption efficiency was calculated to evaluate the U removal ability of NWs/SP‐CSSB gels from simulated radioactive wastewater.

### 4.5 Uranium Removal From Contaminated Real Water Samples

Underground water and potable water were obtained from Haikou of Hainan province, and were spiked with 1000 ppb uranium solutions. NWs/SP‐CSSB gels were immersed with a *m/V* value of 0.13 g L^−1^ (pH 3), and after a certain contact time, some solutions were taken out, and the residual U uranium concentration was determined by using ICP‐MS to analyze the U removal ability from the contaminated underground water and potable water.

## Author Contributions

Z. L., S. G., S. S., and N. W. conceived the research and designed the experiments. Z. L., S. G., and S. S. carried out the experiments, H. W., W. Z., H. W., T. L., and Y. Y. performed a part of characterization tests and uranium removal. All authors took part in the analyses and discussions of the data. S. S., Z. L., S. G., and N. W. wrote and revised the paper.

## Conflicts of Interest

The authors declare no conflicts of interest.

## Supporting information




**Supporting File**: advs76507‐sup‐0001‐SuppMat.docx.

## Data Availability

The data that support the findings of this study are available from the corresponding author upon reasonable request.
