# Supplementary material for: Nanowires Reinforce Oriented Macroporous Aerogels for Rapid Uranium Removal from Acidic Radioactive Wastewater
Source: Adv Sci (Weinh). 2026 Jul 28;13(55):e76507. doi: 10.1002/advs.76507 (PMC13410801; doi:10.1002/advs.76507)
Supplement: Supplementary file 1 [file ADVS-13-e76507-s001.docx]

*Supporting Information for*

**Nanowires Reinforce** **Oriented** **Macroporous Aerogels for Rapid Uranium Removal from** **Acidic Radioactive Wastewater**

Ze Liu^1^, Simin Guan^1^, Se Shi*, Hao Wang, Wenya Zhou, Hui Wang, Tao Liu, Yihui Yuan, Ning Wang*

State Key Laboratory of Marine Resource Utilization in South China Sea, School of Marine Sciences, Hainan University, Haikou 570228, P. R. China

^1^These authors contributed equally to this work.

*Corresponding author

E-mail: shise@hainanu.edu.cn; wangn02@foxmail.com

**Keywords:** uranium removal; rapid rate; nanowires reinforced; directional microchannels; “reinforced concrete” structures

**Experimental section**

**Materials**

Sodium phytate, chitosan, and uranium hexahydrate nitrate [UO_2_(NO_3_)_2_·6H_2_O] were purchased from Macklin Reagent Co., Ltd. 2-(N-3-Sulfopropyl-N, N-dimethyl ammonium) ethyl methacrylate was purchased from Sigma-Aldrich. Oleic acid was purchased from Shanghai Aladdin Biochemical Technology Co., Ltd. Methanol, ethanol, sodium hydroxide, calcium chloride, ammonium persulfate, and acetic acid were purchased from Xilong Scientific Co., Ltd.

**Synthesis of HAP nanowires:** HAP NWs were synthesized by using a solvothermal method ^[1]^. First, NaOH (3.15 g), CaCl_2_ (0.999 g), and NaH_2_PO_4_·2H_2_O (2.82 g) were dissolved in 45 mL of ultrapure water, respectively. Then, these three solutions were sequentially added to a mixture containing 28.08 mL of oleic acid, 14.25 mL of methanol, and 40.5 mL of ultrapure water under magnetic stirring. The mixture was poured into three 100 mL Teflon autoclaves and was heated for 24 h at 180°C. Then, the white slurry products were collected and washed with ethanol and ultrapure water three times, respectively, and the hydroxyapatite nanowires (HAP NWs) were obtained.

**Characterization**

The functional groups of NWs/SP-CSSB were confirmed via FTIR (Nicolet iS10, Thermo Scientific, USA). The microstructure and element distribution mapping were investigated by using scanning electron microscope (Gemini SEM 300, Carl Zeiss, Germany). The pore size distribution information was obtained using physical adsorption apparatus (ASAP 2460, USA). The mechanical strength was tested using a universal tensile machine (LDW-1, Shanghai Songdun, China). The hydrophilicity was studied via a contact angle gauge (JC2000D5, Shanghai Zhongchen, China). The uranium concentration was determined using UV-Vis spectrophotometer (UV1780, Shimadzu) and ICP-MS (I-CAP RQ, Thermo Scientific, USA). The adsorption mechanism was analyzed by using XPS spectroscopy (ESCALAB 250XI, Thermo Fisher, USA).

1 **Supplementary Figures**

**Figure S1** Elemental distribution of the SP-CSSB.

**Figure S2** EDX spectra of the SP-CSSB.

In elemental mapping image and EDX spectra (**Figure S1 and S2**), the appearance of P element (characterization of SP) verified the present of SP (sodium phytate) in the samples, indicating the highly efficient electrostatic bonding of SP with CSSB.

**Figure S3** Comparison of FTIR spectra of CS, SBMA, and CS-SBMA, indicating the successful grafting of SBMA on CS.

**Figure S4** EDS mapping image of CS-SBMA, indicating the successful grafting of SBMA on CS.

**Figure S5** Comparison of water solubility and hydrophilia of the samples before and after polymerization, indicating the successful grafting of SBMA on CS.

**Figure S6** Penetration rate of uranium solutions (pH 3, 12 ppm U-spiked) in NWs/SP-CSSB gels.

Besides the dynamic contact angle determinations by using deionized water, the penetration rate of uranium solutions in NWs/SP-CSSB gels was also measured to evaluate the hydrophilicity during uranium adsorption. As shown in **Figure S6**, uranium solutions also can quickly penetrate the gels (only need 0.033 s), indicating that the prepared gels will possess a rapid solution transportation rate during uranium adsorption.

**Figure S7** Pore size distribution information of NWs/SP-CSSB gels (with NWs) and SP-CSSB gels (without NWs).

First, the apparent density (ρ‌_a_) of NWs/SP-CSSB aerogel was determined via the following equation, and the calculated ρ‌_a_ value was 64.4 mg/cm^3^ for the prepared aerogel, indicating its lightweight characteristics.

$$\rho_{a}=\frac{m}{v}$$

**Figure S8** Digital photo of the lightweight NWs/SP-CSSB aerogels.

Meanwhile, the petals were intact without any collapse (**Figure S8**), intuitively showing their ultralight characteristics of the NWs/SP-CSSB aerogels.

In addition, the porosity of NWs/SP-CSSB aerogels was measured from the apparent and skeletal densities (ρ‌_s_) of the aerogels (as shown in the following equation), and the calculated porosity was 96.3% for the fabricated aerogels. Herein, the skeletal density of the aerogels was determined by using an automatic true density analyzer (Micromeritics AccuPyc Ⅱ 1340, USA).

$$Porosity\left( \% \right)=(1-\frac{\rho_{a}}{\rho_{s}})\times100\%$$

Furtherly, the BET area was determined by using the N_2_ adsorption-desorption experiments. **Figure S9** showed that the specific surface area (5.04 m^2^/g) of NWs/SP-CSSB gels (with nanowires) increased 66.34% compared with that of SP-CSSB gels (without nanowires), indicating that the NWs can improve the microstructures and enhance the contact area between gels and uranium solutions.

**Figure S9** N_2_ adsorption-desorption curves to calculate the BET area of NWs/SP-CSSB gels (with NWs) and SP-CSSB gels (without NWs).

**Figure S10** Photos of NWs/SP-CSSB gels (with NWs) and SP-CSSB gels (without NWs) after being loaded a 200 g weigth and after being immersed in water.

**Figure S11** SEM image of SP-CSSB gels (without NWs) in the X-Z plane.

**Figure S12** Effect of the solution temperature on uranium adsorption for NWs/SP-CSSB gels.

Solution temperature is a key parameter that affects the uranium adsorption performance. The effect of temperature on uranium adsorption efficiency was determined in 12 ppm uranium solutions with pH 3 at m/V of 0.06 g L^-1^. As shown in **Figure S12**, the sorption efficiency increased with the rise of solution temperature, and the sorption efficiency reached 97.4% at 306.15 K. Subsequently, the sorption efficiency was basically unchanged with the increase of solution temperature. Therefore, the optimal temperature was 306.15 K for uranium adsorption of NWs/SP-CSSB gels, and the adsorption experiments were performed at 306.15 K.

**Figure S13** Effect of adsorbent dosage on uranium adsorption for NWs/SP-CSSB gels.

**Figure S14** Pseudo-first-order and pseudo-second-order models for uranium adsorption of NWs/SP-CSSB gels.

**Figure S15** Uranium adsorption capacities of NWs/SP-CSSB gels in the spiked solutions with different uranium concentrations.

**Figure S16** Langmuir and Freundlich models to investigate the uranium adsorption behavior of NWs/SP-CSSB gels.

**Figure S17** Effect of coexisting anions (usually existed in radioactive wastewater) on uranium adsorption for NWs/SP-CSSB gels.

Besides the competing cations, the effect of coexisting anions (usually existed in radioactive wastewater ^[2,3]^) on uranium adsorption efficiency for the prepared gels was also investigated. In the experiments, the concentrations of competing anions were set to 10 times (120 ppm) the initial uranium concentration, respectively. As shown in **Figure S17**, the uranium adsorption efficiencies of gels reached 82.95%~93.49% in the interfering ions of Cl^-^, NO_3_^-^, NO_2_^-^, SO_4_^2-^, and I^-^, respectively, indicating that the NWs/SP-CSSB gels still can efficiently capture U(VI) in the interferences of competing anions.

**Figure S18** Comparison of FTIR of NWs/SP-CSSB gels before and after uranium adsorption.

**Figure S19** Photos of NWs/SP-CSSB gels before and after uranium adsorption.

**Figure S20** EDX spectra of NWs/SP-CSSB gels after uranium adsorption.

EDX spectra of NWs/SP-CSSB gels after uranium adsorption was determined to further investigate the uranium uptake performance. As shown in **Figure S20**, besides the characteristics of SBMA (S element), NWs (Ca element) and SP (P element), a new signal (U element) was significantly present in EDX spectra, indicating that the U(VI) was indeed adsorbed on the gels.

**Figure S21** Photos of the U-loaded NWs/SP-CSSB gels after being immersed in agitated solutions for three days.

The convenient collection of gel was beneficial for its practical application. Therefore, after uranium adsorption, the NWs/SP-CSSB gels were immersed in an agitated solution to assess their structure stability. As shown in **Figure S21**, the gels were intact after the long-term immersion for three days (144 times the adsorption equilibrium time) in the agitated solutions, indicating their good structure stability, and they could be conveniently collected from the solutions after the uranium adsorption.

**Figure S22** Comparison of XPS (in S 2p area) of NWs/SP-CSSB gels before and after uranium adsorption.

2 **Supplementary Tables**

**Table S1** Ionic concentrations of the simulated radioactive wastewater.

| Metal ions | Initial concentration/ppm | Metal ions | Initial concentration/ppm |
| --- | --- | --- | --- |
| U | 18.9 | Co^2+^ | 3.08 |
| Ba^2+^ | 6.18 | Ni^2+^ | 3.30 |
| Mg^2+^ | 1.53 | Zn^2+^ | 3.34 |
| Pb^2+^ | 10.1 | Sr^2+^ | 4.22 |
| Cr^3+^ | 3.25 | Cu^2+^ | 4.18 |
| Al^3+^ | 8.83 | Fe^3+^ | 5.63 |
| Mn^2+^ | 5.50 | Cd^2+^ | 6.21 |

**Table S2** Comparison of the uranium removal efficiency in recent years.

| **Adsorbents** | **Sorption equilibration time (min)** | **pH** | **Remove rate** | **Forms** | **year** | **Reference** |
| --- | --- | --- | --- | --- | --- | --- |
| rGO/ZIF-67 | 48h | 4.01 | 100% | gels | 2020 | [4] |
| X-alginate | 24h | 3 | 98% | gels | 2022 | [5] |
| HAP | 10min | 4 | 99.4% | gels | 2022 | [6] |
| ZIF-67 | 4h | 8.5 | 98.3% | powder | 2023 | [7] |
| Bi/Bi_2_O_3−x_@COFs | 180min | 5 | 93.9% | powder | 2023 | [8] |
| AO-C_3_N_4_ | 120min | 5 | 94.2% | powder | 2023 | [9] |
| GOA | 6h | 5 | 99% | gel | 2024 | [10] |
| PBSAC | 7h | 8 | 96% | powder | 2024 | [11] |
| GG@ZrP | 120min | 6 | 90% | powder | 2024 | [12] |
| GIS | 4h | 6.5 | 94% | powder | 2024 | [13] |
| Tp-Bpy | 180min | 5 | 96.3% | powder | 2024 | [14] |
| CFs-SAA-IEF | 480min | 8 | 90.12% | fiber | 2024 | [15] |
| LZ-P | 90min | 4 | 94% | powder | 2024 | [16] |
| HCPA | 5h | 8.5 | 98% | powder | 2024 | [17] |
| H-UiO-66-PA | 50min | 5 | 94% | powder | 2025 | [18] |
| W_18_O_49_ | 3h | 6 | 92.79% | powder | 2025 | [19] |
| Py-COF | 300min | 5 | 90% | powder | 2025 | [20] |
| Ni@C-700 | 120min | 3-10 | 93.9% | powder | 2025 | [21] |
| PAN-AO/PVA | 8h | 6 | 89.03% | fiber | 2025 | [22] |
| TiO_2_@PCN | 210min | 8 | 95.8% | powder | 2025 | [23] |
| COF-nTs_3_ | 100h | 8 | 93.5% | powder | 2025 | [24] |
| NWs/SP-CSSB gel | 30 min | 3  (acidic wastewater) | 97.39%  (reduce U to 9.75 ppb) | gels  (easily collected) |  | **This work** |

**Table S3** Kinetic parameters for uranium adsorption of NWs/SP-CSSB gels.

| C_o_ (ppm) | Pseudo-second-order | | | Pseudo-first-order | | |
| --- | --- | --- | --- | --- | --- | --- |
|  | *R^2^* | *q*_e_  (mg g^-1^) | *k*_2_  (g (mg min)^-1^) | *R^2^* | *q*_e_  (mg g^-1^) | *k*_1_ (min^−1^) |
| 12 | 0.99994 | 194.93 | 0.01907 | 0.42909 | 21.04 | 0.03667 |

**Table S4** Parameters of Langmuir and Freundlich models for uranium adsorption of NWs/SP-CSSB gels.

| Langmuir | | | Freundlich | | |
| --- | --- | --- | --- | --- | --- |
| R^2^ | q_m_ (mg g^-1^) | K_L_ (L mg^-1^) | R^2^ | K_F_ (mg g^-1^) | 1/n |
| 0.99839 | 483.09 | 1.5109 | 0.82609 | 202.21 | 0.3847 |

3 **References**

[1] Z. C. Xiong, Y. J. Zhu, Z. Y. Wang, Y. Q. Chen, H. P. Yu, *Adv. Funct. Mater.*, **2022**, 32, 2106978.

[2] X. Cao, J. Zhang, J. Pan, Y. Li, Y. Ma, X. Du, L. Feng, B. Huang, Y. Yuan, L. Mao, N. Wang, A. M. Al-Enizi, A. Nafady, S. Ma, *Adv. Sci.*, **2025**, 13, e17224.

[3] M. S. V. Naga Jyothi, S. Gomosta, A. R. Parvathy, S. Baiju, S. M. Maliyekkal, Chem. Eng. J., **2025**, **505**, 159575.

[4] M. Zhao, A. Tesfay Reda, D. Zhang, ACS Omega, **2020**, 5, 8012-8022.

[5] E. Georgiou, G. Raptopoulos, M. Papastergiou, P. Paraskevopoulou, I. Pashalidis, ACS Appl. Polym. Mater., **2022**, 4, 920-928.

[6] T. Xiong, Q. Li, J. Liao, Y. Zhang, W. Zhu, J. Hazard. Mater., **2022**, 423, 127184.

[7] A. Das, D. Roy, J. Pandu, S. De, *Sep. Purif. Technol.*, **2023**, 310, 123137.

[8] X. Liu, R. X. Bi, Z. H. Peng, L. Lei, C. R. Zhang, Q. X. Luo, R. P. Liang, J. D. Qiu, *J. Hazard. Mater.*, **2023**, 455, 131581.

[9] K. Yu, Y. Li, X. Cao, R. Wang, L. Zhou, L. Wu, N. He, J. Lei, D. Fu, T. Chen, R. He, W. Zhu, *J. Hazard. Mater.*, **2023**, 460, 132356.

[10] T. P. Gandhi, M. S. V. Naga Jyothi, S. Gomosta, A. Pamarthi, S. Manna, S. M. Maliyekkal, *J. Clean. Prod.*, **2024**, 435, 140433.

[11] Y. A. Boussouga, J. Joseph, H. Stryhanyuk, H. H. Richnow, A. I. Schäfer, *Water Res.*, **2024**, 249, 120825.

[12] V. Dhanya, N. Rajesh, *Int. J. Biol. Macromol.*, **2024**, 282, 136830.

[13] M. Sobczyk, C. Nguyen Dinh, M. Marzec, E. Bazarkina, K. O. Kvashnina, A. Cwanek, E. Łokas, T. Bajda, *J. Clean. Prod.*, **2024**, 449, 141206.

[14] P. Gao, Y. Hu, Z. Shen, G. Zhao, R. Cai, F. Chu, Z. Ji, X. Wang, X. Huang, *Nat. Commun.*, **2024**, 15, 6700.

[15] B. Li, J. Liu, Q. Liu, J. Zhu, J. Yu, S. Chen, Y. Song, R. Li, J. Wang, *Chem. Eng. J.*, **2024**, 499, 156164.

[16] Z. Xia, C. R. Zhang, X. J. Chen, Y. J. Cai, S. M. Yi, R. P. Liang, J. D. Qiu, *Chem. Eng. Sci.*, **2025**, 301, 120708.

[17] A. Das, A. Jana, D. Das, S. Biswas, H. Seshadri, M. S. Rao, S. De, Ind. Eng. Chem. Res., **2024**, 63, 5845–5862.

[18] K. Tuo, J. Li, Y. Li, C. Liang, C. Shao, W. Hou, C. Fan, Z. Li, S. Pu, Z. Chen, Y. Deng, *Small*, **2025**, 21, 2407272.

[19] C. L. Liu, K. Xuan, Y. P. Guo, H. Jiang, W. J. Ma, X. G. Wang, Z. K. Zhou, J. Li, Y. D. Guo, *Rare Met.*, **2025**, 44, 6786–6800.

[20] R. Cai, Y. Hu, Z. Shen, L. Song, H. Pan, Z. Ji, L. Yin, H. Bao, G. Zhao, Y. Ai, X. Wang, X. Huang, *Small*, **2025**, 21, 2501818.

[21] J. Lei, S. Li, Y. Shen, X. Wang, X. Yang, H. Wu, H. Guo, H. Liu, D. Zhang, S. Wang, *ACS Nano*, **2025**, 19, 31818–31828.

[22] G. Yang, Y. Y. Zhang, Q. H. Zhu, X. Xia, N. Pan, C. Ma, J. Liu, Y. Liu, Y. Qin, Q. Zhang, F. Dong, J. Li, X. Nie, *Adv. Funct. Mater.*, **2025**, 35, 2425281.

[23] Y. Jiang, M. An, T. He, B. He, W. Zhang, J. Liang, H. Mei, W. Zhang, P. Li, Q. Fan, *Small*, **2025**, 22, e11153.

[24] M. Ahmad, X. Mao, K. Zhao, M. U. D. Naik, M. R. Tariq, I. Khan, B. Zhang, *Small*, **2025**, 21, 2501578.
